# The impact of cumulative ecological risk on exercise procrastination among college students: the mediating role of negative self-schema and the moderating role of self-compassion

**DOI:** 10.3389/fpsyg.2026.1818145

**Published:** 2026-04-22

**Authors:** Yawen Shen, Kun Wang, Yue Wang

**Affiliations:** 1 Department of Physical Education, Zhongyuan University of Technology, Zhengzhou, China; 2 School of General Education, Zhengzhou University of Economics and Business, Xinzheng, China; 3 School of Education, Zhengzhou University, Zhengzhou, China

**Keywords:** college students, cumulative ecological risk, exercise procrastination, negative self-schema, self-compassion

## Abstract

**Objective:**

Exercise procrastination is a prevalent issue among contemporary Chinese college students, significantly hindering their comprehensive physical and mental development. From the perspective of social ecological theory, this study aimed to explore the influence mechanism of cumulative ecological risk on college students’ exercise procrastination, with a focus on the mediating role of negative self-schema and the moderating role of self-compassion.

**Methods:**

A convenience sampling method was used to recruit 756 college students from three universities in Henan Province. Data were collected through a set of validated scales, including the Cumulative Ecological Risk Scale, Negative Self-Schema Subscale of the Chinese revised version of the Brief Core Schema Questionnaire (BCSQ), Procrastination in Exercise Scale, and Self-Compassion Scale.

**Results:**

(1) Cumulative ecological risk was significantly positively correlated with exercise procrastination. (2) Negative self-schema partially mediated the relationship between cumulative ecological risk and exercise procrastination. (3) Self-compassion significantly moderated the second half of the mediation pathway: the positive predictive effect of negative self-schema on exercise procrastination was stronger among college students with low self-compassion and weaker among those with high self-compassion.

**Conclusion:**

Cumulative ecological risk can not only directly predict college students’ exercise procrastination but also indirectly affect it through the mediating role of negative self-schema. Moreover, self-compassion can buffer the adverse impact of negative self-schema on exercise procrastination. These findings provide a theoretical basis and practical implications for reducing college students’ exercise procrastination and promoting their physical activity participation.

## 1 Introduction

As a cornerstone of a healthy lifestyle, physical exercise is pivotal for promoting physical and mental well-being (Buecker et al., 2021; Pearce et al., 2022). Extensive evidence confirms that regular physical activity reduces the risk of chronic diseases (e.g., cardiovascular diseases and type 2 diabetes) and alleviates symptoms of anxiety, depression, and other negative emotional states (Pinckard et al., 2019; John-Henderson et al., 2020; Singh et al., 2023; Mahindru et al., 2023). However, contemporary college students, confronted with dual pressures from academics and employment, are devoting progressively less time to physical exercise, leading to increasingly prevalent and severe health issues such as sedentary behavior and obesity (Strain et al., 2024; Bull et al., 2020; Jebeile et al., 2022). For college students, active participation in physical exercise helps build a healthy physique (Huang, 2026), enhance psychological regulation capabilities (Sheng et al., 2024; Wang et al., 2025; Wang et al., 2026), and develop correct sports cognition and behavioral habits during the university period, laying a foundation for lifelong exercise (Antonio et al., 2025). Therefore, identifying the key barriers to college students’ regular exercise is crucial, and exercise procrastination may be a critical variable contributing to insufficient physical activity (Tao et al., 2024; Miao et al., 2024).

Exercise procrastination refers to “the voluntary delay of intended exercise” in the absence of external obstacles (Kelly and Walton, 2021). Compared with general procrastination, domain-specific procrastination may exert more severe negative impacts on health and even exacerbate health risks (Yang et al., 2026). Existing cross-sectional and longitudinal studies have consistently demonstrated that exercise procrastination exerts a significant negative predictive effect on physical activity participation; specifically, higher levels of exercise procrastination are associated with lower volumes of physical activity (Kelly and Walton, 2021; Miao et al., 2025). Exercise procrastination and insufficient physical activity are closely associated with the high incidence of sedentary behavior (Solaro et al., 2025) and obesity (Du M.H. et al., 2025). Statistics show that the prevalence of overweight and obesity among adolescents worldwide has increased by nearly tenfold over the past four decades. By 2030, the prevalence of obesity or being overweight among Chinese adults will reach 70.5%, affecting approximately 810 million people (Zeng et al., 2021; GBD 2021 Adolescent BMI Collaborators, 2025). In addition, the prevalence of sedentary behavior among adolescents in China is not optimistic and shows an increasing trend (Yang et al., 2021). Second, exercise procrastination can induce a series of physical and mental health problems in this population (Farren et al., 2018; Hidayat et al., 2023). Exercise procrastination not only deprives individuals of the emotional regulation benefits of physical exercise; but also, as a maladaptive coping mechanism in itself, is significantly associated with high levels of anxiety, depression, and sleep disorders (Kroese et al., 2014; Sirois, 2014). Therefore, identifying the factors contributing to college students’ exercise procrastination holds important theoretical and practical value for increasing their physical activity frequency and sustaining long-term health behaviors.

### 1.1 Cumulative ecological risk and exercise procrastination

According to social ecological theory, individual development intersects with multiple ecological subsystems, including social, family, school, and peer subsystems, and each of which exerts a certain influence on individual behaviors (Bronfenbrenner, 2000). Cumulative ecological risk refers to multiple and cumulative social-ecological environments that exert negative impacts on individuals, including risks at the family, school, and community levels (Li et al., 2016). This concept highlights the cumulative aggregation of heterogeneous risk factors. This concept emphasizes the characteristic of cumulative aggregation of heterogeneous risk factors; that is, it focuses on the influence of the ecological environment in which an individual lives on their overall health and argues that the accumulation of these factors may lead to adverse outcomes. When multiple risk factors coexist and accumulate, they may interact with each other to produce a stacking effect, thereby increasing the likelihood of adverse outcomes (Li et al., 2016; Yu et al., 2025). Therefore, individual risk factors cannot explain the development of exercise procrastination and must be included in a comprehensive analysis of cumulative factors such as family, school, society, and the individual. In recent years, scholars have focused on the link between cumulative ecological risk and individual behaviors, with a consensus that this risk is generally believed to exert a detrimental effect on exercise behaviors in the Chinese cultural context, where academic pressure and collective living environments may amplify the impact of ecological risk factors (Jin and Wang, 2022; Dong et al., 2022). Previous studies have revealed that cumulative ecological risk negatively predicts physical activity behaviors among junior high school students (Du et al. 2025). Cumulative ecological risk can not only directly exert a negative impact on college students’ exercise adherence but also indirectly affect it negatively by weakening exercise motivation and deteriorating the exercise atmosphere (Li and Saibon, 2025). College students’ exercise behavior is not an isolated individual choice but rather the result of dynamic interactions among multiple ecological subsystems, including family, school, community, and peers. When adverse factors within these subsystems accumulate persistently, the resulting “cumulative risk effect” induces exercise procrastination or avoidance behaviors. The family serves as a crucial context for individual development, with factors such as family socioeconomic status, parenting patterns, rearing styles, and the sports environment potentially exerting a significant influence on adolescents’ engagement in physical activities (Du et al., 2026). Within the school subsystem, irrational physical education curriculum design and the lack of an exercise-oriented atmosphere among peers affect college students’ engagement in physical exercise (Zhang et al., 2025; John et al., 2013; Li and Dong, 2022). At the individual level, detrimental factors such as inadequate self-control and a lack of interest in physical exercise interact with and are reinforced by adverse factors from other subsystems (Du et al., 2023). Chinese university students face enduring challenges in educational reform, which have resulted in an excessive emphasis on academic achievement and graduate employment rates. This situation compels students to withstand multiple pressures from academic, familial, and societal sources. Additionally, China’s distinctive one-child family structure may subject students to heightened familial expectations and psychological burdens. Furthermore, the relatively underdeveloped campus sports culture, coupled with the uneven distribution of sports facilities and curricular resources, may restrict students’ opportunities and motivation to participate in physical exercise. The interaction of these factors is likely to intensify their combined ecological risk, thereby amplifying their influence on exercise procrastination.

### 1.2 The mediating role of negative self-schema

While cumulative ecological risk may contribute to exercise procrastination among college students, the underlying mechanisms, especially pathways such as negative self-schema, remain inadequately explored. Therefore, examining these mediating mechanisms is crucial for enhancing the understanding of this relationship and effectively mitigating exercise procrastination among college students exposed to such risk factors.

Self-schema, a cognitive framework derived from past experiences, organizes and guides the processing of self-relevant information (Lewicki, 1984). These schemas can be either positive or negative. The negative self-schema constitutes a pervasive, unfavorable self-concept, leading individuals to systematically encode, store, and recall self-related information in a biased and negative manner, which subsequently influences their behavioral responses (Humphrey et al., 2021). According to schema theory (Barnaby, 2015), the cumulative effect of various environmental risk factors may expose individuals to adversity and impede the fulfillment of their emotional needs, thereby fostering negative self-schemas. For instance, prior research has identified early traumatic experiences as critical antecedents of negative self-schemas (Chen et al., 2022; Chen et al., 2024). Consequently, cumulative ecological risk stemming from the aggregation of risk factors across ecological subsystems is likely a key predictor of negative self-schemas. When diverse ecological risk factors intersect and amplify to form a suboptimal developmental context, individuals accumulate negative life experiences, which shape negative self-cognitions, bias the cognitive processing of self-relevant information, and ultimately influence their behavior.

Self-schemas regulate individual behavior: positive self-schemas enhance domain-specific behavioral performance, whereas negative self-schemas are associated with negative emotions, behavioral avoidance, and inhibition (Cyranowski and Andersen, 1998). Accumulating evidence confirms a significant link between negative self-schemas, unhealthy behavioral patterns, and adverse psychological states (Liao and Chen, 2006). Among college students, negative self-schemas induce biased negative processing of self-relevant information (Appiah-Kusi et al., 2017). For example, when making decisions, activated negative self-schemas direct attention to negative cues (e.g., fatigue, past exercise failures) while neglecting the positive benefits of exercise (e.g., post-exercise mood improvement). This automatic negative cognition may lead to behavioral avoidance such as exercise procrastination. Thus, we propose that negative self-schemas may play a specific mediating role in the association between cumulative ecological risk and exercise procrastination.

### 1.3 The moderating role of self-compassion

While cumulative ecological risk may induce negative self-schemas and subsequent exercise procrastination, this relationship may not be equally strong for all colleges. The stress-buffering model proposes that positive psychological resources can buffer the negative impact of risk factors on maladaptive outcomes. Self-compassion is one such potential protective factor (Rapoport et al., 2022).

Self-compassion is the ability to encounter oneself with goodwill, accept one’s own suffering, and confront oneself with one’s own mistakes without self-criticism (Neff, 2003a). Moreover, it means to face one’s own suffering openly, regardless of whether it is self-inflicted or caused by others. It also means approaching one’s own weaknesses with kindness, without judgment and critical thoughts, instead of distancing oneself (Neff, 2003b). The positive effect of self-compassion on health-promoting behaviors has long been a core focus in the relevant literature. Studies have indicated that individuals with high self-compassion exhibit greater health awareness, reduced smoking and alcohol consumption, and increased willingness to seek medical assistance when needed (Allen and Leary, 2013). High self-compassion is strongly linked to health consciousness, motivation to avoid unhealthy behavior, and health satisfaction (Terry et al., 2013). Moreover, it is associated with higher self-rated health (Sirois, 2020). Research has demonstrated that self-compassion interventions positively influence the self-regulation of health-promoting behaviors, including healthy eating and physical activity (Biber and Ellis, 2019). Furthermore, self-compassion is strongly associated with intrinsic exercise motivation (Magnus et al., 2010) and adherence to regular physical activity (Allen and Leary, 2013). A meta-analysis demonstrated a moderate negative correlation between self-compassion and procrastination (Sirois, 2014). The underlying mechanism involves fostering adaptive emotional responses to personal flaws, setbacks, and failures, specifically enhancing positive emotions while alleviating negative ones (Sirois et al., 2015). Chen and Lv (2023) also found that self-compassion not only negatively predicts procrastination but also reduces it by decreasing shame and experiential avoidance. A recent study indicated that although individuals with high procrastination exhibited a larger discrepancy between planned and actual sporting activity than those with low procrastination, this effect was moderated by self-compassion (Rapoport et al., 2022). Self-compassion reduces bedtime procrastination by lowering the negative affect. Even after controlling for negative affect, the direct effect of self-compassion on reducing bedtime procrastination remained significant, partly because individuals with higher self-compassion tend to employ healthy emotion regulation strategies to mitigate negative emotions (Sirois et al., 2019). More importantly, individuals’ negative self-schemas are associated with negative self-cognition and low self-esteem. College students with lower levels of self-compassion are more prone to mental health problems and maladjustment, whereas those with higher self-compassion tend to adopt adaptive coping strategies, such as maintaining a stance of kindness and understanding toward their own inadequacies, setbacks, and failures, and utilizing mindfulness to avoid excessive immersion in negative emotions and thoughts. Empirical studies have shown that self-compassion is significantly positively correlated with positive psychological outcomes, such as well-being and life satisfaction (Neff and Germer, 2013; Zipagan and Galvez Tan, 2023). Based on this, self-compassion may indirectly moderate the relationship between cumulative ecological risk and exercise procrastination by alleviating the impact of negative self-schemas.

**Figure 1:**
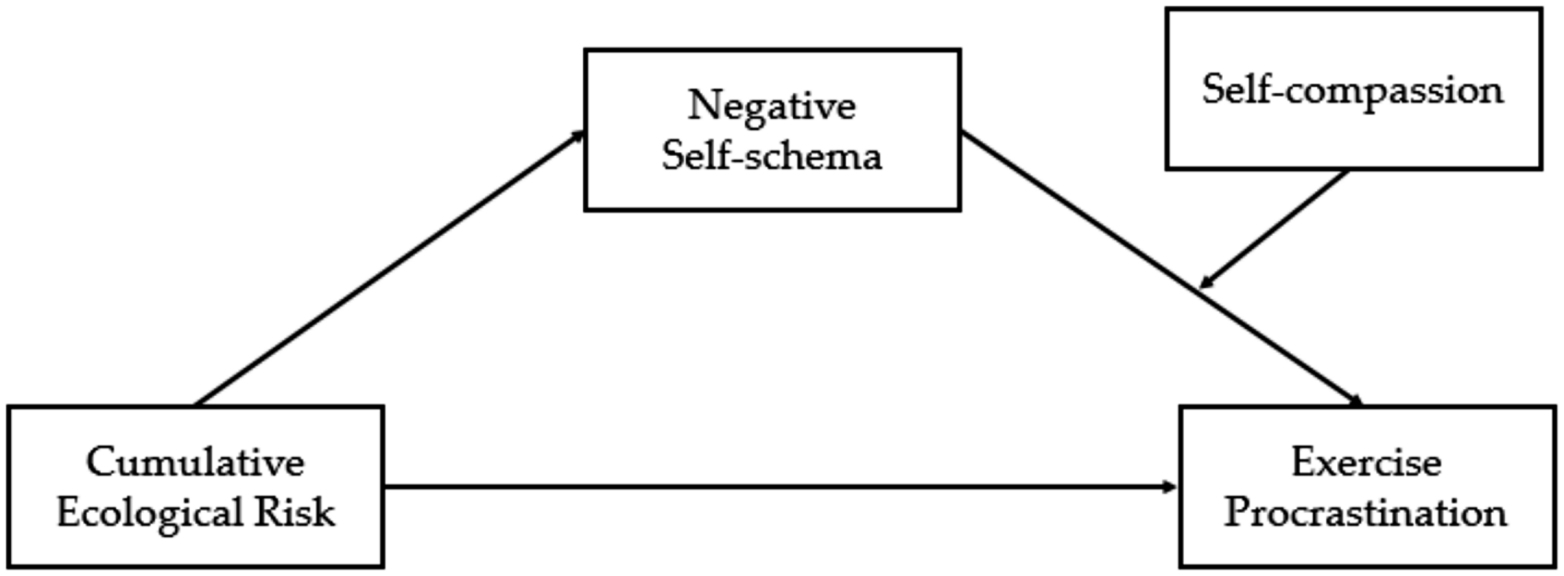
The proposed theoretical model.

To sum up, this study explored the antecedent variables and intrinsic mechanisms of exercise procrastination from the perspective of cumulative ecological risk. Negative self-schemas explain why procrastination occurs, whereas self-compassion addresses when individuals are less likely to procrastinate. Based on previous studies and the theoretical framework, we aimed to investigate both the association between cumulative ecological risk and exercise procrastination among college students and the potential mediating effect of negative self-schemas. The hypothesized model is presented in Figure 1, and the specific research hypotheses are as follows:

*H*1: Cumulative ecological risk positively predicted exercise procrastination.

*H*2: Negative schemas mediate the relationship between cumulative ecological risk and exercise procrastination. Specifically, cumulative ecological risk positively predicted negative schemas, and negative schemas positively predicted exercise procrastination.

*H*3: Self-compassion moderated the relationship between negative self-schema and exercise procrastination. Among college students with high self-compassion, this relationship is weaker.

## 2 Materials and methods

### 2.1 Participants and design

Following the principle of convenience sampling, students from three universities in Henan Province were selected from September to November 2024. The procedure adopted paper-and-pencil group administration for on-site uniform questionnaire collection. The test was conducted with the consent of the physical education (PE) teacher and the participants. The researchers first obtained permission from the PE teachers to access the classes. Subsequently, during the pre-class preparation period, the researchers approached the students, introduced the study’s purpose, and distributed the questionnaires. A collective test was adopted, emphasizing the principles of anonymous and voluntary filling and confidentiality of data. Prior to data collection, oral informed consent was obtained from all participants.

We conducted *a priori* power analysis using G*Power 3.1, with a significance level *α* = 0.05, test efficacy 1-*β* = 0.80, and medium effect size f^2^ = 0.15. The power analysis showed that the minimum required total sample size was 280. A total of 812 college students were recruited for this study. All participants were undergraduate students majoring in fields other than psychology and had no history of psychiatric disorders. Among them, 43.2% were enrolled in humanities or social sciences programs and 56.8% in STEM fields. During the collection of questionnaire responses, 56 invalid questionnaires with regular answers and lack of data were excluded, resulting in a recovery rate of 93.1%. A total of 756 valid questionnaires were obtained from 332 male and 424 female. The average age of the participants was 20.61 ± 1.41 years.

This study adopted a cross-sectional study design to explore the relationship between cumulative ecological risk and exercise procrastination among college students and to verify the proposed theoretical model through correlation and regression analyses.

### 2.2 Measurement

#### 2.2.1 Exercise procrastination

The Procrastination in Exercise Scale was used to measure exercise procrastination (Kelly and Walton, 2021). The Chinese version of this scale has shown good reliability and validity among four different Chinese samples (Miao et al., 2024). The scale includes six items such as “I tend to put exercise off to the next day,” which were rated on 5-point Likert scale from 1 (“completely disagree”) to 5 (“completely agree”). The mean score of all items is calculated, with higher scores indicating higher levels of exercise procrastination. In this study, Cronbach’s alpha was 0.940.

#### 2.2.2 Cumulative ecological risk

Cumulative ecological risk factors were selected based on choices made in previous relevant studies in the field of cumulative ecological risk. Specifically, nine risk factors associated with health-risk behaviors across three environmental systems—family, school, and community—were integrated to construct the Cumulative Ecological Risk Index (Li et al., 2016).

(1) Parental education level: Two items were used to measure the educational levels of the respondents’ father and mother separately, each rated using a six-point scale, from 1 (primary school or below) to 6 (postgraduate or higher). If either parent had a high school education or below (including vocational schools and technical colleges), the response was coded as 1, indicating risk; otherwise, it was coded as 0, indicating no risk.
(2) Family type: Following Dong and Lin (2011), a single item was used to measure family type: “Who are the family members you currently live with?” If the respondent selected the option indicating they do not live with their biological parents, the response was coded as 1, indicating risk; otherwise, it was coded as 0 indicating no risk.
(3) Family socioeconomic status: The Family Economic Stress Questionnaire was adopted to assess the participants’ level of family economic hardship (Wang et al., 2010). This questionnaire consists of 4 items, scored on a 5-point Likert scale, with higher scores indicating a higher level of economic hardship. In the present study, the Cronbach’s *α* coefficient of the scale was 0.853.
(4) The parent–child relationship: It was measured using the Parent–Child Relationship Scale (Bao et al., 2014), which includes items such as, “Are you satisfied with your relationship with your parents?” A 5-point Likert scale was adopted, with higher scores indicating better parent–child relationship quality. In this study, the Cronbach’s *α* coefficient for this scale was 0.77.
(5) Parental relationship: was assessed with two items (Bao et al., 2014) using a 5-point Likert scale. Higher scores indicate better parental relationship.
(6) School connectedness: It was assessed using the School Connectedness Scale (Yu et al., 2011) which includes items such as, “I think I am happy at college.” The scale comprises 6 items, scored on a 5-point Likert scale, with higher scores representing a higher level of school connectedness. In the present study, the Cronbach’s *α* coefficient of the scale was 0.862.
(7) Peer relationships: The Friend Support Subscale of the Perceived Social Support Scale (Wang et al., 1999) was adopted for measurement, which includes items such as, “My friends can share joys and sorrows with me.” This subscale consists of 4 items and uses a 5-point Likert scale for scoring, with higher scores indicating a higher level of friend support. In the present study, the Cronbach’s α coefficient of the subscale reached 0.910.
(8) Deviant peer affiliation: It was assessed using the Deviant Peer Affiliation Questionnaire (Li et al., 2012). This scale uses a 5-point Likert scoring method, with higher scores indicating a higher level of the participants’ association with deviant peers. In this study, the Cronbach’s *α* coefficient for this scale was 0.90.
(9) Peer Victimization: It was measured using the Peer Victimization Questionnaire (Zhou et al., 2013) which includes items such as, “someone argued with you.” The questionnaire consists of 9 items and is scored on a 5-point Likert scale. Higher scores indicating a higher level of peer victimization experienced by the participants. In the present study, the Cronbach’s α coefficient of the scale was 0.848.

For categorical variables, risk was defined as follows: both parents having an educational background of junior high school or below, family structure with non-biological parent care or non-biological parent-grandparent co-care, or poor family economic status; all other cases were classified as no risk. For continuous variables, the P25 or P75 of each risk variable’s score served as the cutoff. Each risk factor was then dichotomously coded, and scores of all risk factors were summed to generate the cumulative ecological risk index. This determination method refers to the standard procedure of the original scale (Li et al., 2016) and is consistent with recent similar studies (Dong et al., 2022), ensuring the rationality of risk classification. A higher index indicated greater cumulative ecological risk in college students. The result of confirmatory factor analysis indicates that the scale has good validity:χ^2^/df = 4.125, RMSEA = 0.057, CFI = 0.932, SRMR = 0.056. The scale exhibited good reliability for the present study, with a Cronbach’s *α* of 0.842.

#### 2.2.3 Negative self-schema

Negative self-schemas among university students were assessed using the Negative Self-Schema Subscale from the Chinese revised version of the Brief Core Schema Questionnaire (BCSQ). This subscale comprises six items, which were originally developed by Fowler et al. (2006) and translated into Chinese by Chen et al. (2022) to ensure cultural adaptability and content validity for Chinese college student samples. For each item, a response of “no” is scored 0. If “yes” is endorsed, participants rate their belief strength on a 4-point scale ranging from “somewhat believe” to “completely believe,” with scores ranging from 1 to 4. Higher total scores reflect a greater level of negative self-schema. In this study, Cronbach’s alpha was 0.867.

#### 2.2.4 Self-compassion

The scale of self-compassion was developed by Neff (2003b) and consists of 26 items, 13 of which are reverse-scored. It has been widely used for the measurement of self-compassion, adopting a 1–5 Likert scale. Higher scores indicate a higher level of self-compassion. The scale comprises six subscales: self-kindness, self-judgment, mindfulness, over-identification, common humanity, and isolation. Chen et al. (2011) conducted reliability and validity analyses on the directly translated Chinese version of this scale. Both exploratory factor analysis (EFA) and confirmatory factor analysis (CFA) supported the six-factor structural model. The Cronbach’s α coefficient (internal consistency reliability) of the scale was 0.84 and the test–retest reliability after 2 weeks was 0.89. The following confirmatory factor analysis results confirmed the validity of the scale: χ2/*df* = 5.174, RMSEA = 0.065, CFI = 0.940, and SRM*R* = 0.047. The Cronbach’s alpha of the scale for the present study was 0.902.

### 2.3 Data analysis

All statistical analyses were performed using IBM SPSS Statistics for Windows. First, we conducted a confirmatory factor analysis to detect significant common method bias. Second, we derived the descriptive statistics for the main research variables and then conducted Pearson’s correlation analysis to examine the relationship between the variables. Third, the SPSS macro program compiled by Hayes in SPSS22.0 was used to verify the mediating role of negative self-schema and the moderating role of self-compassion.

First, we performed descriptive statistics and Pearson’s correlation analyses using SPSS 22.0. Second, to test the hypothesized moderated mediation model, we utilized Hayes’s PROCESS macro (Models 4 and 14) for SPSS. Specifically, Model 4 was used to test the mediating effect of negative schemas on the relationship between cumulative ecological risk and exercise procrastination. Model 14 was used to conduct a moderated mediation analysis to test whether self-compassion moderated the indirect effect of negative schemas. Age and gender were included as control variables.

## 3 Results

### 3.1 Common method bias test

Since all the data in this study came from self-reports of Chinese university students, the results may be affected by common methodological biases. To mitigate this potential issue, prior procedural controls were implemented during study design and data collection, including separating different questionnaires into distinct sections, using reverse scoring for some items, and emphasizing the confidentiality and anonymity of participants’ responses. In addition, the study used the Harman one-way test (Podsakoff et al., 2003) to conduct a post-hoc statistical test for common method bias. The results showed that the characteristic roots of 16 factors were >1, and the first factor could explain 15.274%, which was less than the standard critical value of 40%, thus indicating that there is no considerable common method bias in this study.

### 3.2 Descriptive statistics and correlation analysis

Table 1 presents the means (M), standard deviations (SD), and Pearson correlation coefficients among the main variables. The Pearson correlation analysis showed that cumulative ecological risk was significantly positively associated with Negative self-schema (*r* = 0.343, *p* < 0.01) and exercise procrastination(*r* = 0.426, *p* < 0.001). Cumulative ecological risk had a significantly and negatively association with self-compassion (*r* = −0.210, *p* < 0.01). Self-compassion was negatively associated with both negative self-schema (*r* = −0.308, *p* < 0.01) and exercise procrastination (*r* = −0.375, *p* < 0.01). All correlation directions were consistent with the research hypotheses, providing a preliminary basis for subsequent mediation and moderation analyses.

**Table 1:** Descriptive statistics and correlations among the main variables.

Variables	M(SD)	1	2	3	4
1. Cumulative ecological risk	2.76(1.14)	1			
2. Negative self-schema	1.68(0.39)	0.343**	1		
3. Exercise procrastination	2.89(0.93)	0.426***	0.362**	1	
4. Self-compassion	3.15(0.57)	−0.210**	−0.308**	−0.375**	1

**p < 0.01;***p < 0.001.

### 3.3 Test of the mediating effect of negative self-schema

**Figure 2:**
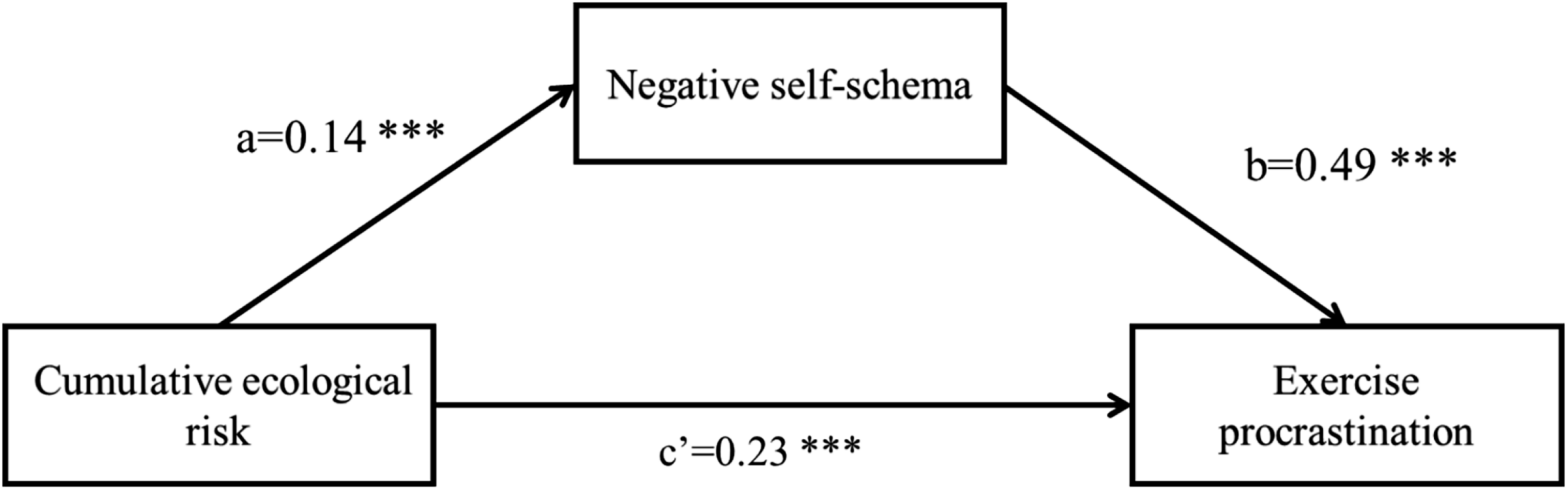
The mediating model of cumulative ecological risk on exercise procrastination via negative self-schema.

The mediating effect of negative self-schema was tested using Model 4 of the PROCESS in SPSS22.0. After controlling for the effects of gender and age, cumulative ecological risk significantly and positively predicted negative self-schema (*β* = 0.14, SE = 0.02, t = 7.65, *p* < 0.001; path a). When both cumulative ecological risk and negative self-schema were included in the regression equation, cumulative ecological risk remained a significant predictor of exercise procrastination (*β* = 0.23, SE = 0.03, t = 7.67, *p* < 0.001; path c’), and negative self-schema also significantly predicted exercise procrastination (*β* = 0.49, SE = 0.02, t = 24.73, *p* < 0.001; path b). A bias-corrected percentile Bootstrap method (5,000 samples) was used to verify the significance of the indirect effect. The results indicated that the mediating effect of negative self-schema between cumulative ecological risk and exercise procrastination was significant, ab = 0.07, Boot SE = 0.02, 95% CI [0.03, 0.11]. The mediated effect accounted for 23.33% of the total effect. These results confirmed that negative self-schema played a significant partial mediating role between cumulative ecological risk and exercise procrastination. The detailed results are presented in Table 2 and Figure 2.

**Table 2:** Bootstrapping indirect effect and 95% confidence interval (CI) for the mediation model.

	Effect	Boot SE	95%CI	Ratio to total effect
Indirect effect	0.07	0.02	[0.03,0.11]	23.33%
Direct effect	0.23	0.03	[0.17,0.29]	76.67%
Total effect	0.30	0.04	[0.22,0.38]	

### 3.4 Test of the moderated mediation effect of self-compassion

We tested the moderated mediation model using PROCESS Model 14, with Cumulative ecological risk served as the independent variable, negative self-schema as the mediator, self-compassion as the moderator, and exercise procrastination as the dependent variable. Table 3 presents the results of the moderated mediation model. Given previous research, gender differs in family ecological risk vulnerability, exercise motivation and persistence, while age corresponds to distinct developmental tasks, leading to varied risk coping strategies and physical activity motivation (Spence et al., 2010; Wang et al., 2024; Martinez and Garcia, 2026; Sheng et al., 2025). Thus, controlling these two variables eliminates confounding effects and ensures accurate testing of the moderated mediation effect. After controlling for gender and age, the interaction term of negative self-schema and self-compassion negatively predicted exercise procrastination (*β* = −0.22, t = −9.38, *p* < 0.001). This suggests that self-compassion moderates the relationship between negative self-schema and exercise procrastination, supporting the moderating effect hypothesis.

**Table 3:** Regression results for the moderated mediation model.

Predictor variables	Negative self-schema			Exercise procrastination		
	*β*	*t*	95%CI	*β*	*t*	95%CI
Gender	0.03	0.42	[−0.05,0.11]	0.05	1.62	[−0.01,0.12]
Age	0.04	1.38	[−0.02,0.10]	0.02	0.36	[−0.06,0.10]
CER	0.14	7.65***	[0.10,0.18]	0.28	6.54***	[0.20,0.36]
Negative self-schema				0.49	14.83***	[0.45,0.53]
Self-compassion	−0.28	−6.84***	[−0.36,-0.20]			
Negative self-schema*self-compassion				−0.22	−9.38***	[−0.26,-0.17]
*R2*	0.44			0.56		
*F*	63.74***			93.12***		

***p < 0.001.

**Figure 3:**
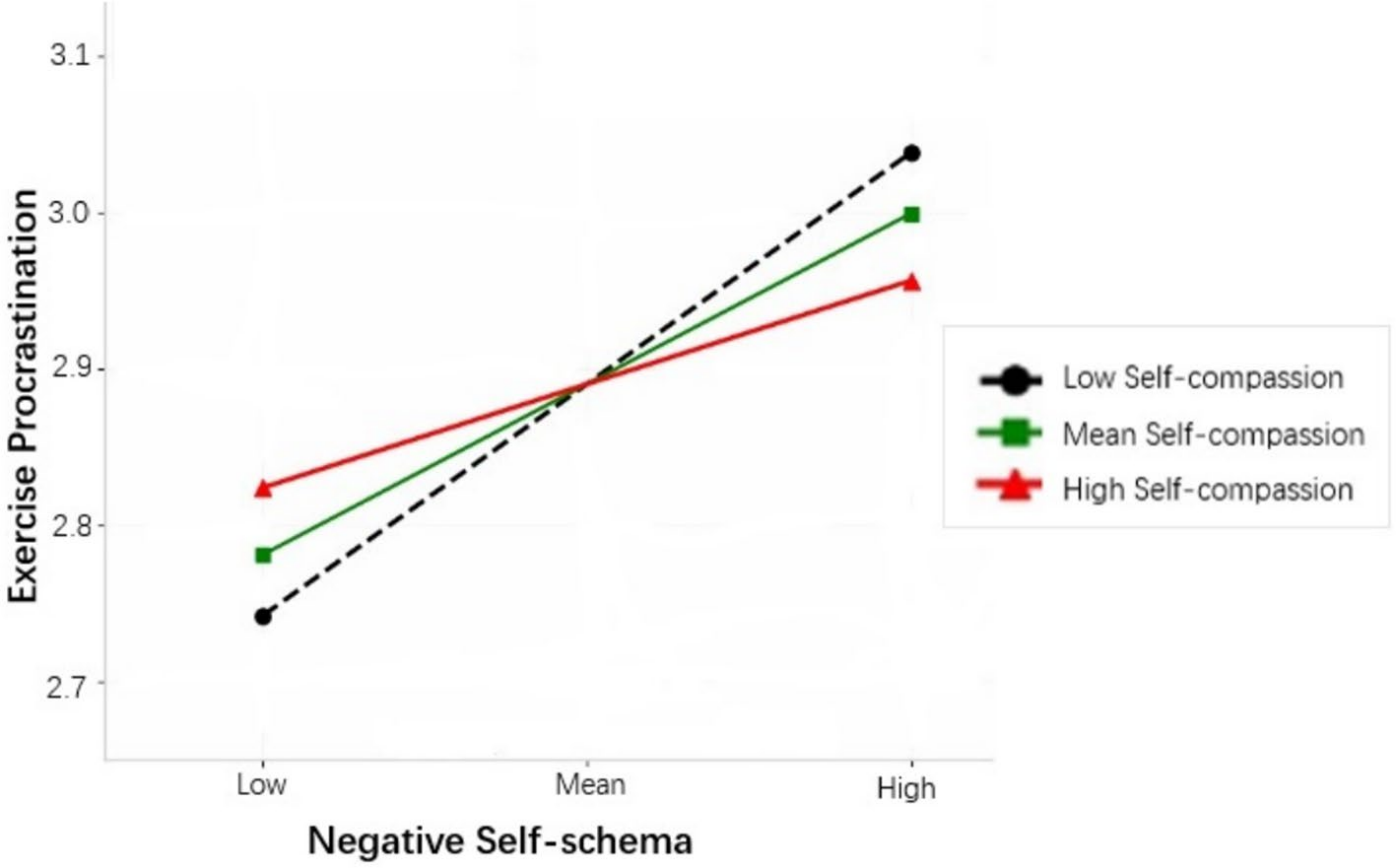
Self-compassion moderating the relationship between negative self-schema and exercise procrastination.

To further explain the moderating effect of self-compassion, self-compassion was divided into high and low groups according to its mean plus or minus one standard deviation (M ± SD), and a simple slope test was performed (see Figure 3). The results showed that negative self-schema was a significant predictor of exercise procrastination when self-compassion scores were low (Effect = 0.38, 95% BootCI [0.28, 0.52]), and a significant but significantly weaker predictor of exercise procrastination when self-compassion scores were high (Effect = 0.17, 95% BootCI [0.05, 0.21]). In other words, the predictive effect of negative self-schema on exercise procrastination diminished as the self-compassion level increased. These results confirmed that self-compassion played a significant negative moderating role in the second stage of the mediation model, supporting Hypothesis 3.

## 4 Discussion

Insufficient physical activity among college students has emerged as a significant public health issue, necessitating the identification of key factors to reverse this trend and enhance their physical fitness. From the perspective of cumulative ecological risk, this study examines its impact on exercise procrastination among college students. Empirical findings indicate that cumulative ecological risk not only directly and positively predicts exercise procrastination but also indirectly exacerbates it by intensifying negative self-schema. Moreover, self-compassion serves as a moderating factor in this process, mitigating the adverse effects of negative self-schema on exercise procrastination and thereby reducing its occurrence.

### 4.1 Cumulative ecological risk and exercise procrastination

In the context of the social-ecological system, various domains such as family, school, community, and peers collectively influence college students’ physical exercise behavior. Factors such as parental rearing styles and sports beliefs, the school sports atmosphere, community sports environment construction, and peer sports interaction relationships are critical environmental drivers for college students’ engagement in physical exercise (Raza et al., 2022; Haidar et al., 2019).

According to social ecological theory, an individual’s behavioral development is embedded in multi-level ecological subsystems. Cumulative ecological risk precisely reflects the scarcity of supportive resources and the prevalence of unstructured socialization characteristics within the multi-layered ecosystems inhabited by college students (Li et al., 2016). When numerous adverse risk factors permeate all ecological domains, college students face challenges in accessing the “comfortable spaces” and positive behavioral support environments necessary for physical exercise (Chen et al., 2025). Such unfavorable ecological environments prompted them to adopt maladaptive behavioral strategies in response to environmental pressures, such as sedentary lifestyles and exercise avoidance (Jin and Wang, 2022; Du et al. 2025). Furthermore, the continuous accumulation of risk factors in family, school, community, and online environments (e.g., insufficient sports venues and facilities, unreasonable opening hours, lack of family sports support, and a weak school sports culture) leads to a gradual decline in the physical and social resources required for exercise. These environmental constraints directly increase the objective barriers for college students to participate in exercise, further inducing behaviors such as “delaying exercise plans” and “avoiding physical exercise,” which serve as important environmental incentives for the emergence of exercise procrastination (Table 4).

**Table 4:** Moderated mediation analysis.

Dependent variable	Moderator	Effect	SE	LLCI	ULCI
Exercise procrastination	-1SD	0.38	0.06	0.28	0.52
	M	0.28	0.05	0.20	0.36
	+1SD	0.17	0.04	0.05	0.21

Bootstrap samples = 5,000; -1SD = Mean-1SD; + 1SD = Mean+1SD. M, Mean; CI, Confidence Interval.

Additionally, cumulative ecological risk depletes individuals’ self-regulatory capacity and cognitive resources through persistent stress exposure. From the perspective of self-regulation theory, prolonged exposure to multiple adverse environmental factors leads to the continuous depletion of individuals’ self-regulatory resources, impairing their self-regulatory capacity and increasing their susceptibility to the “intention-behavior gap” (Miao et al., 2024; Zhang et al., 2024). This mechanism indicates that addressing exercise procrastination cannot rely solely on enhancing individual willpower. Instead, it requires the construction of a low-risk, highly supportive ecological system to mitigate exercise procrastination from both environmental and psychological dimensions, thereby promoting the physical health of college students.

### 4.2 The mediating role of negative self-schema

The findings of this study demonstrate that negative self-schema, as a fundamental cognitive structure, significantly mediates the relationship between cumulative ecological risk and exercise procrastination, acting as a crucial link between environmental factors and individual behavioral development (Bandura, 1986), self-cognition is not innate but is progressively constructed through continuous interaction with one’s ecological environment. Schema theory suggests that environmental factors, such as parenting styles and maternal rumination, influence negative self-schemas (Mcarthur et al., 2019). Cumulative ecological risk, representing an aggregation of adverse factors across multiple ecological systems, serves as a crucial environmental antecedent shaping negative self-schemas among college students (Sutton et al., 2011). When college students are persistently exposed to an ecological environment lacking sports support resources and characterized by frequent negative feedback, their exercise self-efficacy is significantly diminished (Dong et al., 2022). As a result, their self-perception in the realm of physical exercise becomes increasingly negative, leading to the formation of negative self-schemas such as “insufficient athletic ability” and “difficulty achieving exercise goals.”

Once established, negative self-schemas elicit negative expectations of behavioral outcomes. College students with negative self-schemas tend to habitually associate exercise behavior with adverse outcomes such as “failure” and “difficulty.” To avoid anticipated negative experiences, individuals often resort to procrastination behaviors—such as postponing exercise plans or avoiding exercise settings—as a means to alleviate anxiety (Sirois, 2014; Barel et al., 2023). More importantly, negative self-schema diminishes individuals’ exercise self-efficacy, directly reducing their motivation to engage in physical exercise (Stephen and Christopher, 2021). Ultimately, individuals with negative self-schemas may activate cognitive avoidance mechanisms, evading the verification of their negative self-cognition through procrastination, thereby forming a vicious circle of “procrastination – reinforcement of negative self-evaluation – further procrastination” (Zhang et al., 2025). In summary, the mediating role of negative self-schemas in the relationship between cumulative ecological risk and exercise procrastination among college students underscores the interaction between environmental and cognitive factors, facilitating the transmission from “environmental adverse factors” to “psychological cognitive biases” and ultimately to “maladaptive behavioral outcomes.” Understanding this mediating mechanism provides a targeted perspective for the intervention of exercise procrastination: rectifying negative self-schemas in the exercise domain can block the negative transmission of cumulative ecological risk, thereby effectively alleviating exercise procrastination among college students.

### 4.3 The moderating role of self-compassion

This study elucidates the significant moderating role of self-compassion in the relationship between negative self-schema and exercise procrastination, highlighting a notable buffering effect within this association. Specifically, for individuals with high levels of self-compassion, the predictive effect of negative self-schema on exercise procrastination is significantly diminished.

According to self-compassion theory (Neff, 2003a), high self-compassion is characterized by three core components: self-kindness, a sense of common humanity, and mindfulness. Individuals with high self-compassion, when confronted with exercise-related negative self-schemas (e.g., perceptions of “inadequate sports ability” or “failure to accomplish exercise goals”), eschew harsh self-judgment and instead adopt a kind and accepting attitude toward their negative self-cognitions. They recognize such negative perceptions as a universal human experience rather than an individual isolation, and maintain mindfulness to objectively perceive these negative cognitions without over-identifying with the anxiety and frustration they elicit (Neff and Germer, 2013). This cognitive and emotional regulation pattern effectively disrupts the inherent link between negative self-schema and avoidance motivation, thereby reducing the propensity to alleviate negative emotions through exercise procrastination. Conversely, individuals with low self-compassion tend to engage in excessive self-criticism and self-blame when facing negative self-schemas related to physical exercise, leading to psychological isolation due to such negative self-perceptions. This intense negative emotional experience further exacerbates their avoidance of exercise behaviors (Sirois, 2014), making them more likely to engage in procrastinatory behaviors such as delaying exercise plans and avoiding exercise scenarios, thus rendering the predictive effect of negative self-schema on exercise procrastination more pronounced.

Fundamentally, procrastination is defined as a failure of self-regulation, and self-compassion exerts a positive influence on self-regulation, potentially counteracting the adverse effects of procrastination. In the context of physical activity, when individuals experience aversions or negative emotions toward exercise, such feelings may trigger a demand for immediate emotional repair, leading them to deviate from regular physical activity plans and delay the pursuit of long-term goals. Therefore, mitigating exercise procrastination necessitates the enhancement of self-regulatory capacity through self-regulatory resources. Research by Rapoport et al. (2022) has confirmed that self-compassion serves as a self-regulatory resource for coping with exercise procrastination. As a positive psychological resource, self-compassion can modulate individuals’ coping styles toward negative self-schemas, enabling those with high levels of self-compassion to attenuate the adverse effect of negative self-cognition on exercise procrastination.

This finding identifies the boundary condition of negative self-schema’s influence on exercise procrastination and provides a targeted psychological intervention for alleviating college students’ exercise procrastination: cultivating self-compassion can effectively buffer the negative impact of negative self-schema, reduce exercise procrastination stemming from negative cognitive biases, and promote the sustained development of their physical exercise behaviors.

## 5 Implications and limitations

By examining the mechanisms that connect cumulative ecological risk, negative self-schema, self-compassion, and exercise procrastination, this study provides theoretical support and practical implications for addressing exercise procrastination among college students and enhancing their sustained engagement in physical exercise.

Theoretically, this study integrates social–ecological theory and cognitive theory, marking the first examination of the mediating role of negative self-schema between cumulative ecological risk and exercise procrastination, expanding the cognitive pathways linking ecological factors to health behaviors. Additionally, this study identifies the buffering effect of self-compassion as a moderator, addressing the boundary conditions of individual differences in procrastination under ecological risk. Furthermore, it proposes an integrative “environment–cognition–mental resource” framework for exercise procrastination interventions. Collectively, these findings elucidate how ecological factors interact with cognitive representations and positive psychological resources to influence procrastination.

Practically, the findings provide targeted insights for intervening in college students’ exercise procrastination and enhancing their physical exercise participation. We propose the following practical implications consistent with this mechanism. First, constructing a low-risk, high-support ecological environment for college students—by improving sports facilities, enriching campus activities, and strengthening family support—can help prevent the formation of exercise-related negative self-schema. Second, cognitive interventions aimed at reshaping negative self-schema in the exercise context—such as challenging negative self-evaluations and building successful exercise experiences—can mitigate its mediating role. Third, fostering self-compassion through psychological education and group activities can buffer the impact of negative self-cognition by encouraging students to respond to setbacks with understanding rather than self-criticism. By integrating ecological environment optimization, cognitive schema correction, and positive psychological resource cultivation, educators can synergistically reduce cumulative ecological risk and enhance individual psychological quality, effectively promoting a reduction in exercise procrastination and the development of sustained physical exercise behaviors. It is important to note that these suggested intervention approaches, derived from the current correlational findings, would benefit from further validation through longitudinal or intervention studies to establish their causal efficacy in reducing college students’ exercise procrastination.

This study has several limitations that warrant further research. First, the cross-sectional design precludes definitive conclusions on the causal relationships, failing to confirm the dynamic causal chain of ecological risks shaping negative self-schema and leading to exercise procrastination, nor fully reflecting the long-term buffering effect of self-compassion. Second, this study employed convenience sampling, with the sample drawn solely from three universities in Henan Province, which limits the generalizability of the findings. Differences in student population characteristics, levels of exposure to ecological risk, and exercise behavior habits may exist across regions and different types of colleges. Third, while the PROCESS macro was employed to test the moderated mediation model, future research could utilize structural equation modeling to provide a more rigorous representation of the theoretical model and facilitate more robust hypothesis testing. Additionally, this study controlled for only two variables—age and gender—without including additional covariates such as socioeconomic status or academic performance, which may introduce potential confounding. Fourthly, combining self-report scales with objective measurement methods (e.g., behavioral observations, teacher/peer evaluations, sports behavior tracking) could reduce the impact of subjective bias and improve the reliability and validity of the research results. Finally, conducting in-depth research on the structure of cumulative ecological risk, exploring the differential effects of single risk factors and their cumulative effects on exercise procrastination, and clarifying the key ecological risk factors that induce exercise-related negative self-schema is recommended.

## Data Availability

The raw data supporting the conclusions of this article will be made available by the authors, without undue reservation.
