# Supplementary material for: The impact of cumulative ecological risk on exercise procrastination among college students: the mediating role of negative self-schema and the moderating role of self-compassion
Source: Front Psychol. 2026 Apr 22;17:1818145. doi: 10.3389/fpsyg.2026.1818145 (PMC13143838; doi:10.3389/fpsyg.2026.1818145)
Supplement: Supplementary file 1 [file Data_sheet_1.pdf]

Appendix

Procrastination in Exercise Scale

For each of the following statements, please decide whether it applies to you, using the scale provided:

	Strongly disagree	Disagree	Neither agree nor. disagree	Agree	Strongly agree
1. I tend to put exercise off to the next day.	1	2	3	4	5
2. I get distracted by other, more fun things when I am supposed to be working out.	1	2	3	4	5
3. I delay working out.	1	2	3	4	5
4. I often find myself feeling behind on my exercise routines.	1	2	3	4	5
5. I find myself putting off my physical activity, even though I know I should not.	1	2	3	4	5
6. I delay exercising until it is too late to complete my planned workout	1	2	3	4	5

Negative self-schema Scale

Please read each statement carefully and select the option that best reflects your level of belief, where "0" indicates "I do not hold this belief" and "4" indicates, "Believe it totally":

	Do not hold this belief	Believe it slightly	Believe it moderately	Believe it much	Believe it totally
1. I am unloved	0	1	2	3	4
2. I am worthless.	0	1	2	3	4
3. I am weak	0	1	2	3	4
4. I am vulnerable	0	1	2	3	4
5. I am bad	0	1	2	3	4
6. I am a failure	0	1	2	3	4

Self-Compassion Scale (SCS) - Chinese Version

Please read each statement carefully and, based on how you typically feel and think during difficult times, select the option that best reflects your situation. "1" indicates "Almost never" and "5" indicates "Almost always".

	Almost never	Occasionally	About half of the time	Frequently	Almost always
1. I am disapproving and judgmental about my own flaws and inadequacies.	1	2	3	4	5
2. When I am feeling down, I tend to feel	1	2	3	4	5

like most other people are probably happier than I am.					
3. When things are going badly for me, I see the difficulties as part of life that everyone goes through.	1	2	3	4	5
4. When I think about my inadequacies, it tends to make me feel more separate and cut off from the rest of the world	1	2	3	4	5
5. I try to love towards myself when I am feeling emotional pain.	1	2	3	4	5
6. When I fail at something important to me, I become consumed by feelings of inadequacy.	1	2	3	4	5
7. When I am feeling down, I remind myself that there are many other people in the world feeling as I am.	1	2	3	4	5
8. When times are difficult, I tend to be tough on myself.	1	2	3	4	5
9. When something upsets me, I try to keep my emotions in balance.					
10. When I feel inadequate in some way, I try to remind myself that most people share feelings of inadequacy.	1	2	3	4	5
11. I am intolerant and impatient towards those aspects of my personality I do not like.	1	2	3	4	5
12. When I am going through a very hard time, I give myself the caring and tenderness I need.	1	2	3	4	5
13. When I am feeling down, I tend to feel like most other people are probably happier than I am.	1	2	3	4	5
14. When something painful happens, I try to take a balanced view of the situation.	1	2	3	4	5
15. I try to see my failings as part of the human condition.	1	2	3	4	5
16. When I see aspects of myself that I do not like, I get down on myself.	1	2	3	4	5
17. When I fail at something important to me, I try to keep things in perspective.	1	2	3	4	5
18. When I am really struggling, I tend to feel like other people must be having an easier time of it.	1	2	3	4	5
19. I am kind to myself when I am	1	2	3	4	5

experiencing suffering.					
20. When something upsets me, I am carried away with my feelings.	1	2	3	4	5
21. I get down on myself when I feel down.	1	2	3	4	5
22. When I am feeling down, I try to approach my feelings with curiosity and openness.	1	2	3	4	5
23. I am tolerant of my own flaws and inadequacies.	1	2	3	4	5
24. When something painful happens, I tend to blow the incident out of proportion.	1	2	3	4	5
25. When I fail at something that is important to me, I tend to feel alone in my failure.	1	2	3	4	5
26. I try to be understanding and patient towards those aspects of my personality I do not like.	1	2	3	4	5

Cumulative Ecological Risk

Please carefully read each of the following statements and tick "√" in the corresponding option based on your actual situation.

	completely consistent	basically consistent	somewhat consistent	basically inconsistent	completely inconsistent
1. My family cannot afford to buy new clothes.	1	2	3	4	5
2. My family cannot afford to buy a good house/residence	1	2	3	4	5
3. My family cannot afford to buy the food I like.	1	2	3	4	5
4. My family has no extra money for the whole family to engage in recreational activities	1	2	3	4	5
5. When discussing issues together, my parents will consider my opinions.	1	2	3	4	5
6. My parents trust my judgment ability.	1	2	3	4	5
7. When facing important matters, I like to seek my parents' opinions.	1	2	3	4	5
8. When I am angry, my parents	1	2	3	4	5

always try to show understanding.					
9. I will share my problems and troubles with my parents.	1	2	3	4	5
10. I have far more troubles than my parents know.	1	2	3	4	5
11. I receive little attention from my parents.	1	2	3	4	5
12. The relationship between my father and mother is very good.	1	2	3	4	5
13. My father and mother often quarrel.	1	2	3	4	5
14. I prefer to surf the internet or chat with others rather than talk openly with my parents.	1	2	3	4	5
15. I think my parents like to compare me with others, which makes me feel troubled.	1	2	3	4	5
16. My parents are always busy with their own things and have no time for me.	1	2	3	4	5
17. I feel a sense of closeness with the people in my college.	1	2	3	4	5
18. I am glad to be part of my college.	1	2	3	4	5
19. I think I am happy at college.	1	2	3	4	5
20. The school has an infirmary and a psychological counseling room, which can provide timely help when I have health problems.	1	2	3	4	5
21. I usually keep to myself in college.	1	2	3	4	5
22. The teachers in my college treat students fairly.	1	2	3	4	5
23. I feel safe in college.	1	2	3	4	5
24. Teachers care about and support me a lot.	1	2	3	4	5
25. My friends can truly help me.	1	2	3	4	5
26. I can rely on my friends when I encounter difficulties.	1	2	3	4	5
27. My friends can share joys and sorrows with me.	1	2	3	4	5
28. I can discuss my problems with my friends.	1	2	3	4	5

Over the past 12 months, how many of your friends have experienced the following situations? Please tick "√" in the corresponding option.

	None	Few	Some	Most	All
1. Smoked cigarettes	1	2	3	4	5
2. Got drunk from drinking alcohol	1	2	3	4	5
3. Robbed, extorted, or threatened others	1	2	3	4	5
4. Cheated in exams	1	2	3	4	5
5. Stolen things from others or from stores	1	2	3	4	5
6. Been addicted to the internet	1	2	3	4	5
7. Received disciplinary punishment from school	1	2	3	4	5
8. Skipped classes without telling parents or teachers	1	2	3	4	5

In the past 6 months, have you experienced the following things while interacting with your peers? Please tick "√" in the corresponding option.

	None	Rarely	Sometimes	Often	Always
1. Someone yelled at you or called you names	1	2	3	4	5
2. Someone hit you first	1	2	3	4	5
3. Someone bumped into you or pushed you	1	2	3	4	5
4. Someone argued with you	1	2	3	4	5
5. Someone threatened you	1	2	3	4	5
6. Someone tried to control or bully you	1	2	3	4	5
7. Someone made fun of you or teased you	1	2	3	4	5
8. Someone threatened to beat you up	1	2	3	4	5
9. Someone spread rumors about you	1	2	3	4	5
10. Someone has touched my private parts or even done things that harmed me.	1	2	3	4	5
